# Predictive Factors of Recurrence in Patients with Major Depressive Disorder

**DOI:** 10.1192/j.eurpsy.2025.1335

**Published:** 2025-08-26

**Authors:** N. Kissa, N. Ait Bensaid, A. Korchi, F. Laboudi, A. Ouanass

**Affiliations:** 1 University Psychiatric Hospital Arrazi, salé, Morocco

## Abstract

**Introduction:**

Major depressive disorder (MDD) presents as episodes lasting at least two weeks, affecting mood, cognitive, and neurovegetative functions, leading to significant distress [1]. Remission is challenging, with rates of only 30-50% after 6-8 weeks of antidepressant treatment [2]. The STAR*D study shows that 30-40% of patients achieve remission with first-line treatment, and a third fail even after four trials [3]. Recurrence risk is high, reaching 90% after three or more episodes [4][5].

**Objectives:**

The study aims to identify predictive factors for recurrence in major depressive disorder patients at Arrazi Psychiatric Hospital in Salé and to assess the impact of sociodemographic, biological, psychological, socio-environmental, and treatment-related factors on recurrence risk.

**Methods:**

This retrospective, descriptive, and analytical study will collect data from medical records of major depressive disorder patients at Arrazi Psychiatric Hospital in Salé, including sociodemographic, clinical, and therapeutic details. Standardized data extraction ensures anonymity, and factors will be analyzed through univariate, bivariate, and multivariate statistical methods (logistic regressions), with adjusted odds ratios calculated for significant factors and a p-value threshold of < 0.05 applied.

**Results:**

The study shows that women represent approximately 60-70% of patients, with an average age of 30-40 at the first episode and often limited social support in 50-60% of cases. About 40% present psychiatric comorbidities, and 20-30% have physical comorbidities. Nearly 50% of patients have recently experienced stressful events, and 30-40% live in precarious conditions. Therapeutic adherence is insufficient for 30-40% of cases, and 25-35% discontinued treatment prematurely. Meanwhile, those who benefited from combined therapy represent 50-60%, showing a lower recurrence risk.

**Conclusions:**

The recurrence of major depressive disorder poses a major clinical challenge, impacting long-term patient management. Identifying risk factors can enhance preventive interventions and therapeutic follow-up, while an integrative approach considering sociodemographic and psychological factors may help reduce relapse rates. These findings underscore the need for further research into the mechanisms of recurrence in major depressive disorder.

**Disclosure of Interest:**

None Declared

